# Genomic landscape and tumor mutational features of resected preinvasive to invasive lung adenocarcinoma

**DOI:** 10.3389/fonc.2024.1389618

**Published:** 2024-05-13

**Authors:** Yangui Lin, Dan Li, Hongliang Hui, Haoran Miao, Min Luo, Bhaskar Roy, Binbin Chen, Wei Zhang, Di Shao, Di Ma, Yanbing Jie, Fan Qiu, Huaming Li, Bo Jiang

**Affiliations:** ^1^ Department of Thoracic Cardiovascular Surgery, The Eighth Affiliated Hospital of Sun Yat−sen University, Shenzhen, Guangdong, China; ^2^ Community Health Center, The Eighth Affiliated Hospital of Sun Yat-sen University, Shenzhen, Guangdong, China; ^3^ Hangzhou Institute of Medicine (HIM), Chinese Academy of Sciences, Hangzhou, Zhejiang, China; ^4^ BGI Genomics, BGI-Shenzhen, Shenzhen, China; ^5^ Beijing Etown Academy, Beijing, China

**Keywords:** adenocarcinoma *in situ*, minimally invasive adenocarcinoma, invasive adenocarcinoma, genomic landscape, targeted exome sequencing, lung adenocarcinoma

## Abstract

**Introduction:**

Adenocarcinoma *in situ* (AIS) and minimally invasive adenocarcinoma (MIA) are considered pre-invasive forms of lung adenocarcinoma (LUAD) with a 5-year recurrence-free survival of 100%. We investigated genomic profiles in early tumorigenesis and distinguished mutational features of preinvasive to invasive adenocarcinoma (IAC) for early diagnosis.

**Methods:**

Molecular information was obtained from a 689-gene panel in the 90 early-stage LUAD Chinese patients using next-generation sequencing. Gene signatures were identified between pathology subtypes, including AIS/MIA (n=31) and IAC (n=59) in this cohort. Mutational and clinicopathological information was also obtained from the Cancer Genome Atlas (TCGA) as a comparison cohort.

**Results:**

A higher mutation frequency of *TP53*, *RBM10*, *MUC1*, *CSMD*, *MED1*, *LRP1B*, *GLI1*, *MAP3K*, and *RYR2* was observed in the IAC than in the AIS/MIA group. The AIS/MIA group showed higher mutation frequencies of *ERBB2*, *BRAF*, *GRIN2A*, and *RB1*. Comparable mutation rates for mutually exclusive genes (*EGFR* and *KRAS*) across cohorts highlight the critical transition to invasive LUAD. Compared with the TCGA cohort, *EGFR, KRAS, TP53*, and *RBM10* were frequently mutated in both cohorts. Despite limited gene mutation overlap between cohorts, we observed variant mutation types in invasive LUAD. Additionally, the tumor mutation burden (TMB) values were significantly lower in the AIS/MIA group than in the IAC group in both the Chinese cohort (P=0.0053) and TCGA cohort (P<0.01).

**Conclusion:**

These findings highlight the importance of distinguishing preinvasive from invasive LUAD in the early stages of LUAD and both pathology and molecular features in clinical practice, revealing genomic tumor heterogeneity and population differences.

## 1 Introduction

Lung cancer remains the leading cause of cancer-related deaths and ranks second in newly diagnosed carcinoma worldwide, with 2.2 million new cases and 1.8 million new deaths per year (1). Non-small-cell lung cancer (NSCLC) accounts for 80%~85% of all lung cancers, and it is composed of squamous-cell carcinoma, adenocarcinoma, and large-cell carcinoma (1). LUAD is a major subtype of lung cancer. Lung adenocarcinoma (LUAD) accounts for approximately 40% of all lung carcinomas (1). If diagnosed and treated in the early stages, the survival rate for lung cancer participants who undergo complete surgical resection within 1 month after diagnosis can reach 92% (2). Furthermore, the overall survival (OS) rate varies among different stages of primary lung cancer; stage IA (tumor size < 1 cm) has a 5-year OS rate of 82%, whereas stages beyond IIB have a 5-year OS rates of less than 50% (3).

Most cases of early-stage lung cancer are treated with surgery alone, whereas combination therapy may be used in other scenarios (4). The 2015 World Health Organization (WHO) classification of lung tumors introduces new concepts for LUAD, such as adenocarcinoma *in situ* (AIS), minimally invasive adenocarcinoma (MIA) (5); Invasive adenocarcinoma (IAC) also recommended by the WHO to be classified by comprehensive histologic pattern, which includes five predominant adenocarcinoma patterns: lepidic, acinar, papillary, solid, and micropapillary (5). Others include invasive mucinous adenocarcinoma, colloid adenocarcinoma, fetal adenocarcinoma, and enteric adenocarcinoma (5). The recurrence-free survival (RFS) rate varies among different pathology subtypes, with AIS and MIA having a 100% RFS rate (6). There is a 40% recurrence rate in NSCLC cases with all pathology subtypes after complete primary tumor resection and systematic lymph node dissection within 5 years (7). Clinical judgment and treatment therapy for early-stage lung cancer is crucial in clinical practice. Early diagnosis of pathology subtypes, along with an understanding of the genetic landscape, can help to prolong the survival of lung cancer patients.

In recent years, mutation profiles of NSCLC have been clarified, with frequent mutations identified in driver genes such as epidermal growth factor receptor (*EGFR*), and erb-b2 receptor tyrosine kinase 2 (*ERBB2*) (8, 9). Various treatment options are available for patients with NSCLC, including targeted therapy, immunotherapy, chemoradiotherapy (CRT), and complete resection (4, 10). However, the crucial predictive biomarkers for early-stage NSCLC remain largely unknown and require validation and reproducibility (11). Genomic intratumor heterogeneity has been identified in early-stage LUAD, and different pathology subtypes, including AIS (12). In preinvasive and invasive LUAD, *EGFR* mutation is the most common driver alterations across AIS, MIA, and IAC (13). In addition, other canonical cancer gene mutations such as *ERBB2*, NRAS proto-oncogene, GTPase (*NRAS*), and B-Raf proto-oncogene (*BRAF*) are early trunk mutations during the carcinogenesis of LUAD (13, 14). Meanwhile, mutations in the tumor protein p53 gene (*TP53*) and cell mobility, gap junction, and metastasis-related genes may be late events associated with subclonal diversification and neoplastic progression (14).

Targeted exome sequencing has been widely used for mutation detection in cancer therapy (15). In one retrospective study, four gene mutations were identified, including *MAP2K1* insertion-deletions (indels), *BRAF* non-V600E kinase mutations, and exon 20 insertions (20ins) in both *EGFR* and *ERBB2*, which were enriched in pre-invasive tumors among 3,254 Chinese patients with LUAD (16). The dataset from TCGA has also been used for comparison studies or validation of the gene signature results (17). Despite these observations, genomic characteristics and ethnic differences in the WHO 2015 pathology classifications of LUAD genomics have yet to be systematically elucidated because of the lack of a sufficiently large cohort.

To address the challenges mentioned above, our study aimed to identify the unique mutational features that distinguish preinvasive from invasive LUAD in both our Chinese cohort and the TCGA cohort. To achieve this, we collected epidemiological and clinicopathological information from early-stage LUAD patients with AIS/MIA or IAC from both our Chinese cohort and the TCGA cohort. In addition, surgically resected specimens were subjected to targeted exome sequencing to reveal the molecular status differences between AIS/MIA and IAC.

## 2 Materials and methods

### 2.1 Patients and sample collection

The resected tissue samples were collected from 90 primary LUAD patients who underwent resection surgery from July 2020 to December 2021. Gene mutation data was obtained using a capture-based targeted sequencing approach with a 689-gene panel. Relevant epidemiological and clinicopathologic information records were also obtained. The public dataset of TCGA was derived from the MSK study with histologic data in cBioportal for Cancer Genomics (18). Only patients data from stages I and II were included and the clinical data was selected following the 2015 WHO criteria (5).

### 2.2 DNA Extraction, library preparation, and target capture sequencing

Genomic DNA (50-200 ng) was extracted from formalin-fixed paraffin-embedded (FFPE) samples using QIAamp DNA FFPE Tissue Kit (Qiagen, Hilden, Germany). The extracted tissue DNA samples were qualified and used to construct cDNA libraries (Integrated DNA Technologies, Coralville, IA, USA). Custom-designed 689-gene probe panels (Integrated DNA Technologies, Coralville, IA, USA) were used to capture their respective, as listed in **Supplementary Table S1**. To eliminate germline mutations, a control library was created using DNA extracted from peripheral blood samples. The cDNA libraries were sequenced using the MGISEQ-2000 platform (MGI, Shenzhen, China) by the manufacturer’s recommendations. The workflow for potentially actionable variants was introduced following a previous pipeline (19).

### 2.3 Sequencing data analysis and immunohistochemistry

The raw fastq data generated by the MGISEQ-2000 sequencer underwent filtering by SOAPnuke (RRID : SCR_015025, https://scicrunch.org/resolver/SCR_015025) to remove reads with low quality. The reference human genome used was GRCh37/hg19, and clean reads were mapped to this genome using the UCSC genome browser (RRID : SCR_005780, https://scicrunch.org/resolver/SCR_005780). Single nucleotide variants (SNVs) and small insertions/deletions (Ins/Del) were identified using the Genome Analysis Tool kit (GATK) with parameters adapted to HaloPlex-generated sequences. Copy number variants (CNVs) were called using the CNVnator read-depth algorithm. Tumor mutation burden (TMB) was assessed through targeted sequencing of approximately 1.25Mb, which broadly recapitulated previous results of whole exome TMB analysis. Tumor mutation burden was calculated as the number of all nonsynonymous mutations per 0.7 Mb of the targeted coding region. TMB was obtained by calculating the number of mutations (allele frequency > 1.5%) in non-driver genes per Mb in each sample. MSIsensor and MANTIS were used to detect the status of microsatellite instability (MSI) (20, 21).

Programmed death ligand 1 (PD-L1) expression was conducted by immunohistochemistry in the central laboratory. The PD-L1 expression was determined by the Tumor Proportion Score (TPS) method with the IHC 22C3 pharmDx kit (Agilent Technologies, Santa Clara, California, USA).

### 2.4 Statistical analysis

Visualization and statistical analyses were performed using R (v4.1.0) and prism graphpad (RRID : SCR_000306, https://scicrunch.org/resolver/SCR_000306). All distributed data were presented as mean ± standard deviation. Chi-square test was employed to analyze the differences between AIS/MIA and IAC groups. P < 0.05 is regarded as statistically significant. GO and KEGG were performed using Database for Annotation, Visualization, and Integrated Discovery v6.8 (DAVID, https://david.ncifcrf.gov/) (22). DAVID v6.8 (https://david.ncifcrf.gov/tools.jsp), an online set of functional annotation tools, was used to analyze biological processes, cellular components, molecular functions, and pathways for DEGs. GO terms and KEGG pathways (RRID : SCR_012773, https://scicrunch.org/resolver/SCR_012773) with P value <0.05 were considered statistically significant.

## 3 Results

### 3.1 Clinical characteristics and mutation spectrum of LUAD patients

Clinical data were employed for 90 primary LUAD patients. The mean age was 59 years (standard deviation [SD] = 12.9 years) for this cohort, with 42.2% (38/90) male and 58.7% (52/90) female. 13.3% (12/90) were adenocarcinoma in stage 0, Others were 67.8% (61/90) in stage I, and 18.9% (17/90) in stage II (**Supplementary Table S2**). The detailed clinical characteristics of the patients are shown in **Table 1** and **Supplementary Table S2**. In brief, 12.2% (11/90) had Ex19Del mutant *EGFR*, 34.4% (31/90) were L858R mutant *EGFR*, others 12.2% (11/90) were absent of these two classical *EGFR* activating mutations. The whole LUAD patients were also divided into subgroups of AIS, MIA, and IAC. The age of the AIS/MIA group was significantly younger than the IAC group (52.4 versus 62.5 years, P < 0.001). Besides, the composition of sex and tumor stage were also significantly different between AIS/MIA and IAC group patients. The AIS/MIA group had more females and more cancers in the early stages. Other factors such as N stage, MSI, and *EGFR* mutation status (Ex19Del, L858R, Absent) had no difference between the AIS/MIA and IAC groups (**Table 1**).

**Table 1:** Clinical characteristics of 90 patients with primary LUAD.

		Group		
Characteristics	Overall Patients (n = 90)	Patients with AIS/MIA (n = 31)	Patients with IAC (n = 59)	P value
**Age at operation**				<0.001
Mean	59.0	52.4	62.5	
SD	12.9	12.1	12.1	
**Sex, n (%)**				0.0220
Male	38 (42.2%)	8 (25.8%)	30 (50.8%)	
Female	52 (57.8%)	23 (74.2%)	29 (49.2%)	
**EGFR mutation, n (%)**				0.7230
Ex19Del	11 (12.2%)	3 (9.7%)	8 (13.6%)	
L858R	31 (34.4%)	11 (35.5%)	20 (33.9%)	
Absent	11 (12.2%)	4 (12.9%)	7 (11.9%)	
**N status, n (%)**				0.0660
N0	84 (93.3%)	31 (100%)	53 (89.8%)	
N1	6 (6.7%)	0 (0%)	6 (10.2%)	
**MSI status**				0.5770
MSI-H	2 (2.9%)	0 (0.0%)	2 (4.3%)	
MSI-L	5 (7.1%)	2 (8.7%)	3 (6.4%)	
MSS	63 (90.0%)	21 (91.3%)	42 (89.4%)	
**Tumor stage, n (%)**				<0.001
Stage 0	12 (13.3%)	12 (38.7%)	0 (0%)	
Stage I	61 (67.8%)	17 (54.8%)	44 (74.6%)	
Stage II	17 (18.9%)	2 (6.5%)	15 (25.4%)	

AIS, adenocarcinoma *in situ*; IAC, invasive adenocarcinoma; LUAD, lung adenocarcinoma; MIA, minimally invasive adenocarcinoma; MSI, microsatellite instability; MSI-H, high microsatellite instability; MSI-L, low microsatellite instability; MSS, microsatellite stability; SD, standard deviation.

**Figure 1:**
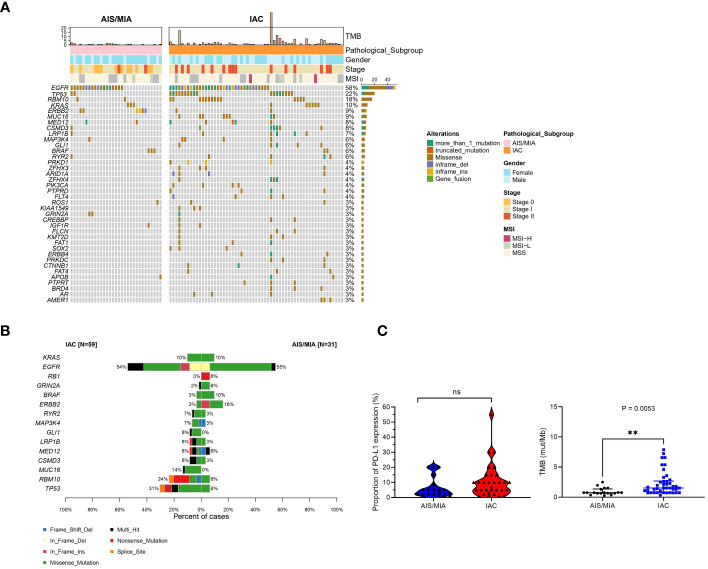
Distinct mutation spectrum of preinvasive and invasive LUAD patients. **(A)** Waterfall plot with top 38 genes for LUAD patients in AIS/MIA and IAC group; **(B)** The mutation types of 15 high-frequent mutated genes between AIS/MIA and IAC; **(C)** Proportion of PD-L1 expression level in AIS/MIA and IAC group (left), Proportion of TMB values in AIS/MIA and IAC group (right). AIS: adenocarcinoma in situ, MIA: minimally invasive adenocarcinoma, IAC: Invasive adenocarcinoma, MSI: microsatellite instability, TMB: tumor mutation burden, ns, not significant, **: P < 0.01.

A panel of 689 cancer-related genes was applied to 90 primary lung tissue samples. Mutation genes were also revealed in this cohort (**Supplementary Table S3**). All 90 patients had at least one potentially actionable variant. Full details of variants for all patients are available in **Supplementary Table S3**. In summary, gene landscape was different between AIS/MIA and IAC groups (**Figure 1A**, **Supplementary Figures S1A, B**). The most frequently mutated genes with various pathology subtypes (AIS/MIA/IAC) are displayed. The mutation frequencies of *TP53* (31% versus 6%), RNA binding motif protein 10 (*RBM10*, 24% versus 6%), mucin-16 (*MUC16*, 14% versus 0%), CUB and sushi multiple domains 3 (*CSMD3*, 8% versus 3%), mediator complex subunit 12 (*MED12*, 8% versus 6%), low-density lipoprotein receptor-related protein 1b (*LRP1B*, 8% versus 3%), GLI family zinc finger 1 (*GLI1*, 8% versus 0%), mitogen-activated protein kinase kinase 4 (*MAP3K4*, 7% versus 3%), and ryanodine receptor 2 (*RYR2*, 7% versus 3%) were higher in the IAC group than in the AIS/MIA group. The AIS/MIA group showed higher mutation frequencies of *ERBB2* (16% versus 3%), *BRAF* (10% versus 3%), glutamate ionotropic receptor NMDA type subunit 2A (*GRIN2A*, 6% versus 2%), and RB transcriptional corepressor 1 (*RB1*, 6% versus 0%) than the IAC groups (**Figure 1A**, **Supplementary Figures S1A, B**). The mutation frequencies were similar between AIS/MIA and IAC groups for the two top driver genes of EGFR (55% versus 54%) and KRAS proto-oncogene, GTPase, (*KRAS*, 10% versus 10%) (**Figure 1A**, **Supplementary Figures S1A, B**). The data suggest that *ERBB2, BRAF, GRIN2A, RB1* were early-stage events for LUAD. The late events were mutations for *TP53, RBM10, MUC1, CSMD3, MED1, LRP1B, GLI1, RYR2* mutations. Besides, *EGFR* and *KRAS* mutations accompanied all the events. In addition, by analyzing the mutation types of 15 high-frequent mutated genes between AIS/MIA and IAC (**Figure 1B**), we identified that the mutation type of *EGFR* varied in IAC, with genes such as *RYR2*, *GLI1*, *LRP1B*, *MED12*, *CSMD3*, *MUC16*, *RBM10*, *TP53* having higher mutation frequency and more mutation types, such as structure change for genes in invasive LUAD.

### 3.2 Identification of core genes in tumors originating from the lung

**Figure 2:**
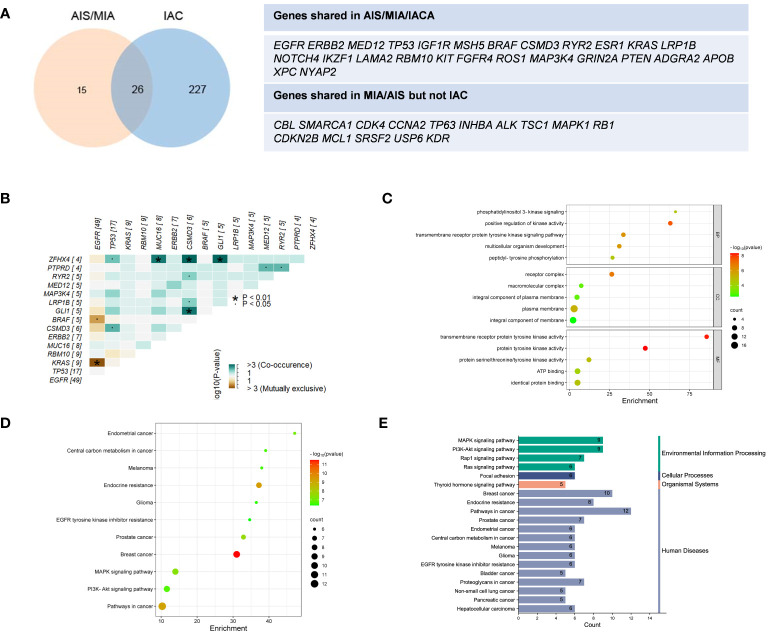
Distinct distribution of genes and pathway analysis. **(A)** Shared genes between IAS/MIA and IAC groups; **(B)** Gene co-occurrence figure for the top 15 frequently mutated genes; **(C–E)** The Gene Ontology (GO) and Kyoto Encyclopedia of Genes and Genomes (KEGG) pathway enrichment for the shared genes between IAS/MIA and IAC groups. ▪: P < 0.05. *: P < 0.01.

In **Figure 1C**, tumor mutation burden (TMB) values of the AIS/MIA group were significantly lower than those of the IAC group (3.92 versus 0.94 mut/Mb, P = 0.0053). The proportion of PD-L1 expression in the IAC group was slightly higher than that in the AIS/MIA group, but this difference was not significant. Furthermore, as shown in **Figure 2A**, these two groups shared 26 genes in common, including *EGFR, ERBB2, MED12, TP53*, insulin-like growth factor 1 receptor (*IGF1R*), mutS homolog 5 (*MSH5*), *BRAF*, *CSMD3*, *RYR2*, estrogen receptor 1 (*ESR1*), *KRAS*, *LRP1B*, notch receptor 4 (*NOTCH4*), IKAROS family zinc finger 1 (*IKZF1*), laminin subunit alpha 2 (*LAMA2*), *RBM10*, KIT proto-oncogene, receptor tyrosine kinase (*KIT*), fibroblast growth factor receptor 4 (*FGFR4*), ROS proto-oncogene 1, receptor tyrosine kinase (*ROS1*), *MAP3K4*, *GRIN2A*, phosphatase and tensin homolog (*PTEN*), adhesion G protein-coupled receptor A2 (*ADGRA2*), apolipoprotein B (*APOB*), XPC complex subunit, DNA damage recognition and repair factor (*XPC*), neuronal tyrosine-phosphorylated phosphoinositide-3-kinase adaptor 2 (*NYAP2*). In addition, *EGFR, ERBB2, MED12, TP53, BRAF*, and *KRAS* are six genes shared across AIS/MIA/IAC groups (**Supplementary Figure S2A**), all of which play an important role during tumorigenesis. There were 15 genes unique to MIA/AIS rather than AIS. More detailed information can be found in **Supplementary Table S3**.

According to the gene co-occurrence figure (**Figure 2B**), there was a significant exclusivity between mutations in *KRAS* (P < 0.01), *BRAF* (P < 0.05), and *EGFR*. Additionally, *CSMD3* was found to co-occur with *TP53, ZFHX4, RYR2, LRP1B* and *GLI1*. Previous studies have shown that high expression of CSMD3 is associated with poor outcomes (23). In our results, we found that zinc finger homeobox 4 (*ZFHX4*) co-occurs with *TP53, MUC16, CSMD3*, and *GL1.* It is worth noting that ZFHX4 was found to be upregulated in lung adenocarcinoma (LUAD) and was associated with a poor prognosis, as indicated by previous research (24).

In the analysis of core genes and related signaling pathways, we adopted the Gene Ontology (GO) and Kyoto Encyclopedia of Genes and Genomes (KEGG) pathway enrichment analysis (25). A total of 20 important pathways in the lung cancer population were grouped (**Figures 2C–E**). The genes shared between preinvasive and invasive LUAD play important roles in the cellular component of the receptor complex, positive regulation of kinase activity for biological processes, and transmembrane receptor protein kinase activity function. Moreover, these genes function in focal adhesion and pathways in cancer, which are crucial for neoplastic progression. Thus, membrane-related kinase activity could be an early event from preinvasive to invasive LUAD. The differentially mutated genes suggest a better understanding of early lung cancer progression.

### 3.3 Clinical implications and comparison with the TCGA cohort

**Figure 3:**
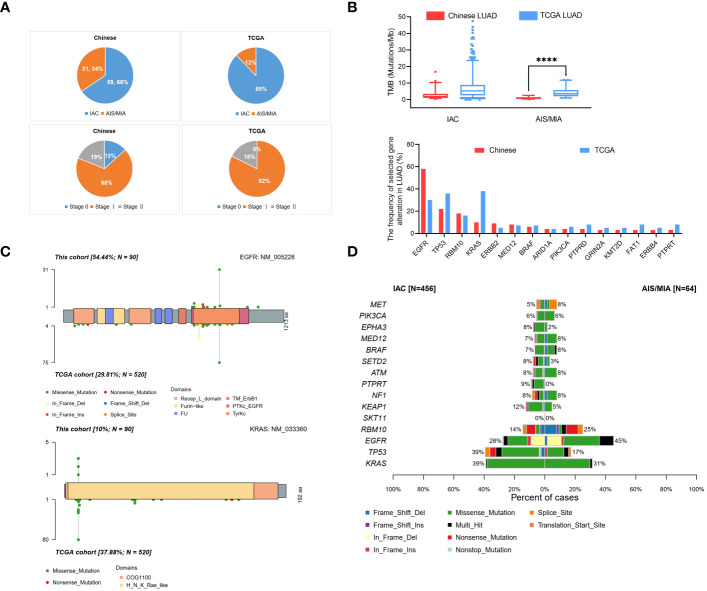
Comparison of data in Chinese LUAD and the TCGA LUAD cohorts. **(A)** Pathology and tumor stage status in our cohort with Chinese patients and TCGA cohort; **(B)** Proportion of TMB values in AIS/MIA and IAC group in our group and TCGA group (up panel), gene alternations in our group and TCGA group (lower panel); **(C)** Distribution and comparison of deleterious mutations detected in EGFR gene (up panel) and KRAS gene (lower panel) both in Chinese group and TCGA group; **(D)** The comparative bar plot of the top 15 frequently mutated genes in the TCGA cohort. ****: P < 0.0001.

To further compare the clinical significance in multiple dimensions, we used the public database from TCGA. Full details of variants for all patients are available in **Supplementary Table S4**. From our Chinese cohort and TCGA cohort, we identified that our Chinese group contained 34% (31/90) AIS/MIA patients, compared with 12% (64/524) in the TCGA cohort. In our Chinese cohort, 66% (59/90) of the patients had IAC, whereas a higher percentage of patients (88%, 460/524) had IAC in the TCGA cohort (**Figure 3A**). Moreover, the tumor stages we analyzed were also different between our cohort and the TCGA cohort. The percentages were 13% (12/90), 68% (61/90), 19% (17/90) for Stage 0/I/II in our cohort, compared with 0% (0/524), 82% (432/524), 18% (92/524) in the TCGA group (**Figure 3A**). TMB values were also analyzed for the Chinese group and the TCGA group (**Figure 3B**, up panel). In the TCGA cohort, the TMB values were significantly higher in the IAC group than in the MIA group (7.625 versus 4.450, P < 0.01). Additionally, it seems that our group had a lower TMB of the AIS/MIA group than the TCGA (0.939 versus 4.450, P < 0.0001). This might be because our group had 13% Stage 0 and we had 12 AIS patients, while the TCGA group was in higher stages with MIA.

By analyzing the mutation spectrum of the top 35 genes in our Chinese cohort and the TCGA cohort, we identified that different genes varied significantly under different circumstances (**Supplementary Figure S2B** and **Figure 3B**, lower panel). We also compared two driver genes, *EGFR* and *KRAS*, in the whole population. The variation frequency of *EGFR* was 54.44% in our cohort, compared with 29.81% in the TCGA cohort (**Figure 3C**, up panel). Additionally, the variation frequency of *KRAS* was 10% in our cohort, compared with 37.88% in the TCGA cohort (**Figure 3C**, lower panel). In **Figure 3D**, the comparative bar plot shows the 10 mutation types of the top 15 genes in TCGA cohort. Although the overlapped genes between our Chinese cohort and TCGA cohort varied, the mutation types increased in invasive LUAD in the TCGA cohort.

## 4 Discussion

We profiled this functional study on 90 early-stage LUAD patients, combining the AIS and MIA groups due to their high 5-year RFS of 100% (6). This RFS was higher than NSCLC cases with all pathology subtypes, which experienced a recurrence rate of 40% within 5 years (7). We analyzed gene mutational profiles using capture-based targeted sequencing and compared the most frequently mutated genes within AIS/MIA and IAC groups. Our results showed that the gene landscape was different between the two groups. The mutation frequency of *TP53*, *RBM10*, *MUC16*, *CSMD3*, *MED12*, *LRP1B*, *GLI1*, *MAP3K4*, *RYR2* was higher in the IAC group than in the AIS/MIA group. Additionally, during the follow-up, there were only two recurrence events in the IAC group (2/25, 8%) and no events in the preinvasive group (0/9, 0%), indicating a difference in recurrence rates between preinvasive and invasive LUAD groups. Although the difference was not significant, this may be due to the short follow-up time of less than 3 years, which was too short for early-stage LUAD.

Genomic intratumor heterogeneity has been identified in early-stage LUAD with different pathology subtypes, including AIS (12, 13). Mutations including *MAP2K1*, *BRAF*, *EGFR*, and *ERBB2*, were identified as enriched in pre-invasive tumors (16). Additionally, *ERBB2*, *NRAS*, and *BRAF* are considered to be early trunk mutations during LUAD carcinogenesis (13, 14). Our study also identified that *ERBB2* and *BRAF* were early-stage events for LUAD with higher mutation frequency in the preinvasive group. *ERBB2* is a proto-oncogene, and it’s alternation is associated with poor survival and worse outcomes (26). Our results also showed that mutations in *BRAF* (P < 0.05) with *EGFR* were significantly mutually exclusive. This has also been recently confirmed in another Chinese cohort (27). In our study, *GRIN2A* and *RB1* were also highly mutated in the preinvasive group and were considered to be early-stage events for LUAD in our Chinese cohort. The mutation frequency of *ERBB2, BRAF, GRIN2A*, and *RB1* was found to be low in the TCGA cohort, and there was no significant difference in mutation frequency between the preinvasive and invasive LUAD groups in TCGA.

In our study, *TP53*, *RBM10*, *MUC16*, *CSMD3*, *MED1*, *LRP1B*, *GLI1*, and *RYR2* were identified as late events with a higher mutation frequency in the invasive LUAD group. *TP53* mutations were the most enriched alternations in invasive LUAD, suggesting that it may play a more important role in the acquisition of invasiveness. It has also been revealed that *TP53* and cell mobility, gap junction, and metastasis-related genes may be late events associated with subclonal diversification and neoplastic progression (13, 14). One study identified that *RBM10* plays an important role in invasive LUAD, whereas another study showed that this gene is significantly mutated in the pre/minimally invasive group (14). It has been reported that as a tumor suppressor gene, *RBM10* can also promote lung cancer (28). For the TCGA cohort results, the mutation frequency of *RBM10* was 16.1%, and it was more highly mutated in the preinvasive group than in the invasive LUAD group (25% versus 14%). Interestingly, MUC16 (CA125) is cleaved and shed into the bloodstream, and its serum level is thought to be a key indicator of lung cancer metastasis to the liver (29). Additionally, *MUC16* plays crucial roles in lung cancer pathogenesis, progression, and chemoresistance (30). In general, *MUC16* might play an important role during the entire process from preinvasive to invasive LUAD.

In both preinvasive and early invasive LUAD, *EGFR* mutation is the most common driver alterations across AIS, MIA, and IAC (13). EGFR is a transmembrane protein that can transduce important growth factor signaling from the extracellular milieu to the cell, thus regulating downstream events including increased proliferation, angiogenesis, metastasis, and decreased apoptosis (31). *KRAS* alterations occur early in the carcinogenesis process and promote cancer cell survival, invasion, and migration (32). Our results showed that mutations in *KRAS* and *EGFR* were mutually exclusive (P < 0.01). This has been validated in another study that *EGFR* mutations are never found in LUAD tumors with *KRAS* mutations (33). In our study, for driver genes such as *EGFR* and *KRAS*, the mutation was similar between the AIS/MIA and IAC groups, indicating that *EGFR* and *KRAS* mutations were separately occurring and accompanying all events. It has also been reported that *EGFR* and *KRAS* mutations are significantly mutated genes in the pre/minimally invasive group (14). In addition, the *EGFR* mutation type varies greatly in IAC compared with AIS/MIA in our Chinese cohort. The distribution and comparison of deleterious mutations detected for *EGFR* and *KRAS* exhibited some overlap between the Chinese group and TCGA group but also showed variations. In our study, the pre-invasive LUAD exhibited a high frequency of driver mutations, including those in *EGFR, KRAS, ALK*, and *ERBB2* genes, which was also found in other publications (14, 34). The population for TCGA cohort also yielded a high incidence of *EGFR* mutations, which might due to the background of the United States (35).

In conclusion, we demonstrated that *ERBB2*, *BRAF*, *GRIN2*, *RB1*, *TP53*, *RBM10*, *MUC1*, *CSMD3*, *MED1*, *LRP1B*, *GLI1*, *MAP3K4*, *RYR2*, *EGFR*, and *KRAS* genes all play crucial roles in carcinogenesis from preinvasive to invasive LUAD in our Chinese cohort. The usability of targeted sequencing for biomarker detection has strong clinical significance for LUAD. Furthermore, in comparison of our findings with the TCGA database, *EGFR, KRAS, TP53*, and *RBM10* were the most frequently mutated genes in the two cohorts. In addition, we also observed an increase in mutation types in IAC in both cohorts. Overall, our current understanding of pathological and molecular features in clinical practice reveals genomic tumor heterogeneity of resectable LUAD.

## Data availability statement

The datasets presented in this study can be found in online repositories. The names of the repository/repositories and accession number(s) can be found in the article/**Supplementary Material**.

## Ethics statement

The studies involving humans were approved by Ethics Committee of The Eighth Affiliated Hospital of Sun Yat-sen University and informed consents from all patient. The studies were conducted in accordance with the local legislation and institutional requirements. The participants provided their written informed consent to participate in this study. The animal study was approved by Ethics Committee of The Eighth Affiliated Hospital of Sun Yat-sen University and informed consents from all patient. The study was conducted in accordance with the local legislation and institutional requirements. Written informed consent was obtained from the individual(s) for the publication of any potentially identifiable images or data included in this article.

## Author contributions

YL: Writing – original draft. DL: Writing – original draft. HH: Methodology, Writing – review & editing. HM: Data curation, Formal analysis, Writing – original draft. ML: Data curation, Formal analysis, Writing – original draft. BR: Writing – review & editing. BC: Data curation, Formal analysis, Writing – original draft. WZ: Data curation, Formal analysis, Writing – review & editing. DS: Data curation, Formal analysis, Writing – original draft, Writing – review & editing. DM: Data curation, Formal analysis, Writing – original draft, Writing – review & editing. YJ: Writing – original draft, Writing – review & editing. FQ: Conceptualization, Writing – original draft. HL: Conceptualization, Writing – review & editing, Supervision. BJ: Conceptualization, Funding acquisition, Resources, Writing – review & editing.

## Supplementary material

Supplementary Figure 1 Mutation spectrum of the LUAD patients in a Chinese cohort. **(A)** Waterfall plot with top frequently mutated genes in LUAD patients with AIS/MIA; **(B)** Waterfall plot with top frequently mutated genes in LUAD patients with IAC.

Supplementary Figure 2 Comparison of data in Chinese LUAD and the TCGA cohorts. **(A)** Shared genes between IAS, MIA and IAC groups in a Chinese cohort; **(B)** Top 35 frequently mutated genes in our Chinese cohort and TCGA cohort.
