# Supplementary figures and images for: Genomic landscape and tumor mutational features of resected preinvasive to invasive lung adenocarcinoma
Source: Front Oncol. 2024 May 13;14:1389618. doi: 10.3389/fonc.2024.1389618 (PMC11128541; doi:10.3389/fonc.2024.1389618)

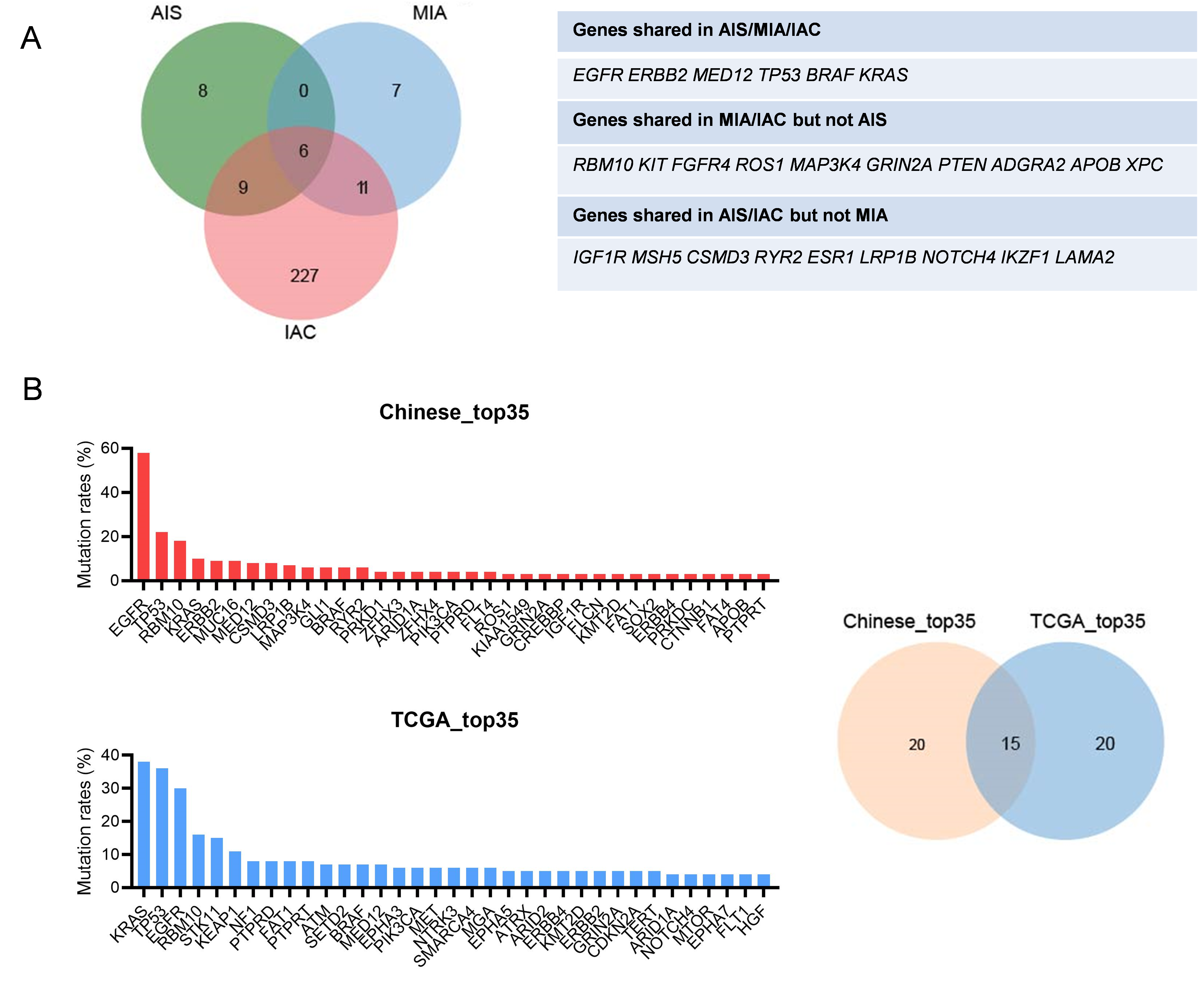

Supplement: Supplementary Figure 1 — Mutation spectrum of the LUAD patients in a Chinese cohort. (A) Waterfall plot with top frequently mutated genes in LUAD patients with AIS/MIA; (B) Waterfall plot with top frequently mutated genes in LUAD patients with IAC. [file Image_1.tif]

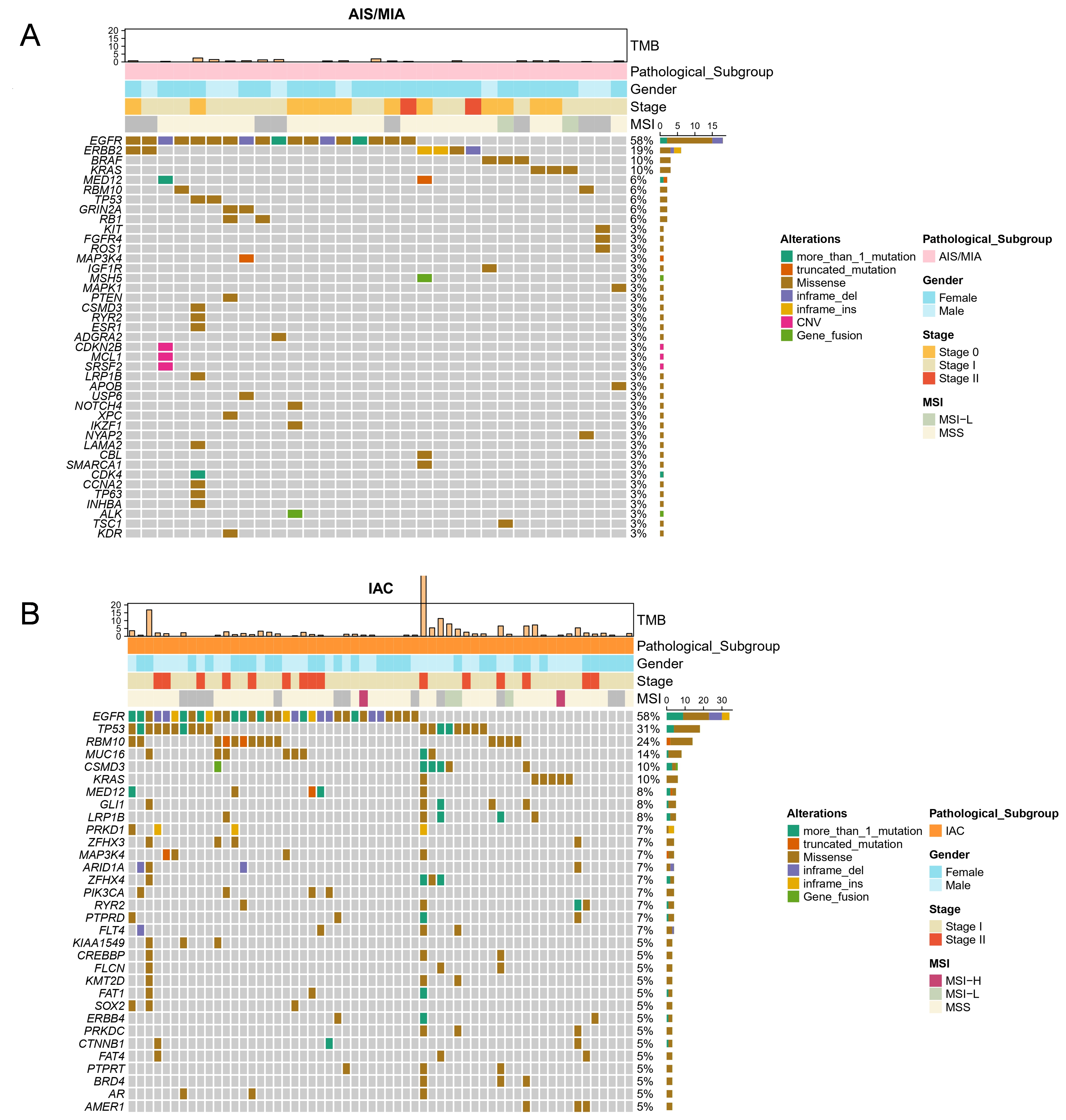

Supplement: Supplementary Figure 2 — Comparison of data in Chinese LUAD and the TCGA cohorts. (A) Shared genes between IAS, MIA and IAC groups in a Chinese cohort; (B) Top 35 frequently mutated genes in our Chinese cohort and TCGA cohort. [file Image_2.tif]
